# Auxin metabolism rates and implications for plant development

**DOI:** 10.3389/fpls.2015.00150

**Published:** 2015-03-17

**Authors:** Eric M. Kramer, Ethan M. Ackelsberg

**Affiliations:** Bard College at Simon's RockGreat Barrington, MA, USA

**Keywords:** auxin biosynthesis, GH3 family, YUCCA family, auxin conjugation, TAA1, oxIAA, IAAsp

## Abstract

Studies of auxin metabolism rarely express their results as a metabolic rate, although the data obtained would often permit such a calculation to be made. We analyze data from 31 previously published papers to quantify the rates of auxin biosynthesis, conjugation, conjugate hydrolysis, and catabolism in seed plants. Most metabolic pathways have rates in the range 10 nM/h–1 μM/h, with the exception of auxin conjugation, which has rates as high as ~100 μM/h. The high rates of conjugation suggest that auxin metabolic sinks may be very small, perhaps as small as a single cell. By contrast, the relatively low rate of auxin biosynthesis requires plants to conserve and recycle auxin during long-distance transport. The consequences for plant development are discussed.

## Introduction

The plant hormone auxin plays a role in virtually every aspect of plant growth and development (Davies, 2004). It is transported cell-to-cell via several families of auxin-specific protein carriers, most famously the PIN family of auxin efflux carriers (Zazimalova et al., 2010). Auxin transport has been the subject of hundreds of studies, and is perhaps the best-characterized example of hormone transport in plants (Kramer et al., 2011). The coordinated spatial organization of auxin carriers determines the sites of auxin accumulation and depletion in a tissue, which in turn signal a wide range of tissue-specific developmental events. The efficiency of auxin transport, and the dramatic phenotypes of some carrier mutants, has led to a widespread view of auxin signaling that tends to overlook the important and complementary role of auxin metabolism.

The last decade has seen remarkable progress in our understanding of auxin metabolism. In particular, many of the gene families involved in auxin biosynthesis, catabolism, conjugation, and conjugate hydrolysis, have now been characterized (Normanly, 2010; Zhao, 2012; Korasick et al., 2013; Ljung, 2013). While the qualitative features of the auxin metabolic pathways are now well-known, there is very limited information available on their quantitative aspects. To better understand the complex interplay of auxin metabolism and transport in plant development, we are obligated to consider not just *which* gene families play a role in auxin metabolism, but *how much* auxin they can synthesize or catabolize per unit time. Studies of auxin metabolism routinely measure the concentrations of auxin and related metabolites, but these results are rarely expressed as a rate that would permit a comparison with the effects of auxin transport [although see (Epstein et al., 1980) for an interesting early example].

The remainder of this paper is organized as follows. The section Preliminaries reviews the metabolic pathways of interest and provides an introduction to the units of metabolic rate. The section Auxin Metabolic Rates presents the main results of our analysis and describes how the metabolic rates were determined. The section Auxin Sinks reviews several examples from plant development where transport-driven sinks accumulate auxin, and speculates that auxin conjugation may help regulate auxin levels in these cases. The section Auxin Metabolism and Transport discusses the interplay of auxin metabolism and transport in the context of auxin sources and auxin transport pathways. The paper concludes in the section Discussion. Further details of our metabolic rate analysis, and tables summarizing all results, appear in the Supplemental Information for this paper.

## Preliminaries

### The metabolic pathways

**Figure 1:**
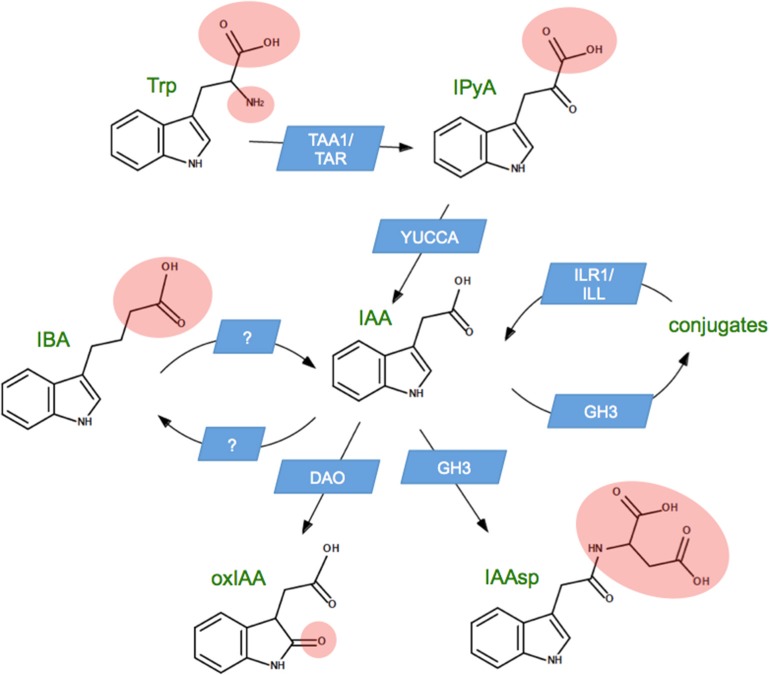
**Principle auxin metabolic pathways in seed plants**. Auxin (indole-3-acetic acid, IAA) is synthesized from tryptophan (Trp) via indole-3-pyruvic acid (IPyA), and undergoes irreversible catabolism via oxindole-3-acetic acid (oxIAA). Auxin can be reversibly conjugated to a wide variety of compounds, generally considered to be storage forms of IAA. Conjugation with aspartic acid to make indole-3-acetyl aspartic acid (IAAsp) is irreversible, so this constitutes a second catabolic pathway. The figure also shows reversible conversion to indole-3-butyric acid (IBA), an auxin-like compound with a role in adventitious root formation. Blue boxes indicate the name of the gene family responsible for each pathway. Pink ellipses are added for clarity, to indicate the portion of each chemical structure that differs from IAA.

Figure 1 illustrates the best-characterized auxin metabolic pathways in seed plants. Biosynthesis proceeds from tryptophan via the gene families TAA1/TAR and YUCCA (Cheng et al., 2006; Stepanova et al., 2008), which convert tryptophan to indole-3-pyruvic acid and then to auxin (indole-3-acetic acid, IAA). The oxidative conversion of auxin to oxindole-3-acetic acid (oxIAA) happens through the action of a protein that has not yet been characterized in eudicots, but which is expected to be a member of the OsDAO gene family recently characterized in rice (Zhao et al., 2013). In addition to these, there are numerous pathways in which auxin is reversibly converted to another form. The most well-known such pathway involves the conjugation of IAA to amino acids by the GH3 gene family (Staswick et al., 2005). Most amino acid conjugates can be hydrolyzed by the ILR1/ILL gene family to release free IAA (LeClere et al., 2002), so that conjugates are sometimes called “storage forms” or “bound” auxin. An exception to this rule is conjugation with aspartic acid to produce indole-3-acetyl-aspartate (IAAsp). IAAsp is not hydrolyzed by plants, so that IAAsp appears to be a second pathway for auxin catabolism (Bialek et al., 1983; Ostin et al., 1998). Figure 1 also shows the inter-conversion between auxin and indole-3-butyric acid (IBA), an auxin-like molecule with several endogenous roles in the plant (Strader et al., 2010), but best known as an exogenous treatment used to promote adventitious root growth (Ludwig-Muller et al., 2005). In the case of IBA metabolism, the gene families remain unknown.

### Units

Metabolic rates are expressed per volume of plant tissue, with units of μM IAA metabolized per hour. Since auxin content is typically reported per gram fresh weight (ng IAA/gFW or nmol IAA/gFW), we make the conversion to μM using the density of the plant material. In cases where the density is not available, we approximate the density of plant tissue using the density of water. This approximation is accurate to ±30% (Iversen, 1949; Ngonyamo-Majee et al., 2008).

### Time scales

In addition to calculating the metabolic rates, where possible we estimate a time scale for metabolism. We quantify biosynthesis by calculating an auxin *replacement time*. This is the time it would take biosynthesis to replace the entire endogenous auxin content already present in the tissue, calculated by dividing the auxin concentration by the rate. This calculation assumes that auxin biosynthesis does not depend sensitively on the auxin concentration. Similarly, for auxin conjugation and catabolism, we compile data on the auxin *half-life*—the time it would take for auxin content to be reduced by 50% at the current rate of auxin metabolism. These calculations, detailed further in the Supplement, assume that auxin conjugation and catabolism are first-order, i.e., the rate is proportional to the auxin content (Barratt et al., 1999). The reader should keep in mind that catabolism and conjugation are usually measured with the application of exogenous auxin, so that rates will tend to be elevated above their endogenous values. In addition, these calculations do not include the feedback mechanisms that regulate auxin concentration in plant tissues (Korasick et al., 2013; Ljung, 2013). Thus, replacement times and half-lives should be regarded as approximate.

## Auxin metabolic rates

We performed a re-analysis of published auxin metabolism data from 31 papers, covering 18 species of seed plants. Before presenting the main results, we present several illustrative examples of our analysis. A detailed description of all analyses performed may be found in the Supplemental Information.

As a first example, we consider the experiment by Ljung et al. (2001) on whole *Arabidopsis thaliana* seedlings. Plants 10 dag were incubated in liquid media with or without added naphthylphthalamic acid (NPA, an auxin transport inhibitor) for 24 h. They used gas chromatography-mass spectroscopy (GC-MS) to quantify the auxin content of leaves during the experiment (their Figure 5). Control leaves maintain a steady auxin content of ~10 pg/mg FW. Leaves from the NPA-treated plants show an increase in auxin content between 8 and 16 h after treatment, from 10 to 22 pg/mg FW. Since NPA is an auxin transport inhibitor, it is unlikely that this increase is due to auxin transport from other parts of the plant. An increase of 12 pg/mg FW in 8 h corresponds to an auxin biosynthesis rate of 8.6 nM/h.

A second example is the biosynthesis of auxin in the apex of the *Zea mays* coleoptile, as estimated from the data of Mori et al. (2005). The authors place excised 2 mm apices on agar and monitor auxin export over several hours using GC-MS. In the first 3.5 h, the apex exports 1470 pg IAA (their Figure 4). Since the total auxin content in the apex is only about 130 pg (their Table 1), and does not vary much during the experiment, we can conclude that the apex is synthesizing 1470 pg/(3.5 h) = 420 pg/h. Dividing by the mass of the apex (Nishimura et al., 2006) and approximating the density of the coleoptile by that of water gives a rate of 1.3 μM/h. The same research group subsequently showed that synthesis in the apex is principally from tryptophan (Nishimura et al., 2009).

As a third example, we consider the results of Sztein et al. (1995), a thorough study of auxin metabolism across the plant kingdom. They placed excised plant organs in 154 μM ^14^C-IAA, then used chromatography to identify many of the most abundant metabolites. For excised Arabidopsis leaves, they found a net metabolite production rate of 11 μg/gFW in 22 h, or 2.8 μM/h, with most of the products in the form of IAA conjugates.

These examples help to illustrate the typical limitations of our estimates for auxin metabolic rates. The determination of biosynthesis rates generally involve tissue excision or a change in growth conditions, while catabolic rates generally involve the application of exogenous auxin. Thus, rate determinations rely on perturbations of the plant that may alter the endogenous metabolic pathways (Tam et al., 2000). However, the qualitative consistency of our data, often calculated using several different techniques, gives us confidence in the main results.

**Figure 2:**
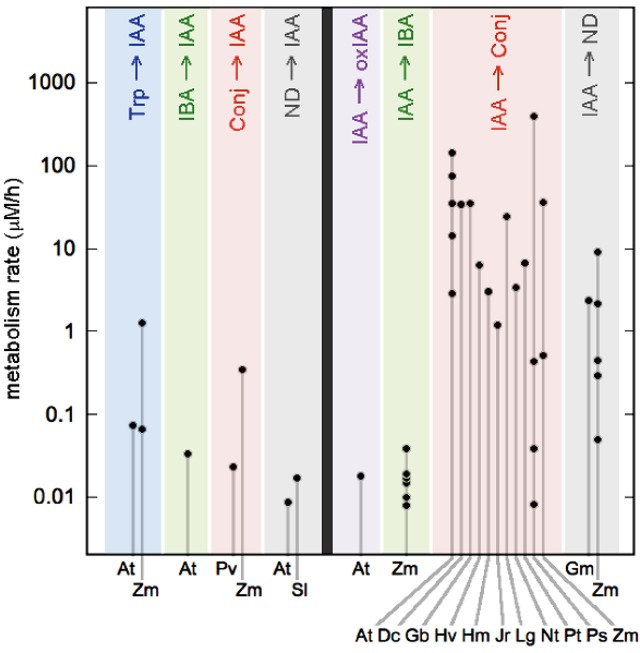
**Metabolic rates in seed plants**. Metabolic pathways are distinguished by color and labeled at top with abbreviations introduced in Figure 1 (Conj, conjugates; ND, not determined). The left half of the figure shows metabolic pathways that add auxin to the tissue. The right half shows pathways that remove auxin. Abbreviations along the bottom indicate the various species for which data is available: At, *Arabidopsis thaliana*; Dc, *Daucus carota*; Gb, *Gingko biloba*; Gm, *Glycine max*; Hv, *Hippuris vulgaris*; Hm, *Hyoscyamus muticus*; Jr, *Juglans regia*; Lg, *Lemna gibba*; Nt, *Nicotiana tabacum*; Pt, *Pinus thunbergiana*; Ps, *Pisum sativum;* Pv, *Phaseolus vulgaris*; Sl, *Solanum lycopersicum*; and Zm, *Zea mays*. Rates are derived from published papers, as summarized in the main text and described fully in the Supplemental Information. Error bars (not shown) are ±30%.

The results of our analysis for auxin biosynthesis and conjugate hydrolysis rates are summarized in the left half of Figure 2 and detailed in the Supplement (Table S1). We draw on data from seven papers, covering one monocot (*Zea mays*) and three eudicots. The eudicot values are all less than 100 nM/h and have a median value of 23 nM/h. By contrast, *Zea mays* tissues have biosynthesis and hydrolysis rates an order of magnitude larger than those observed in eudicots. There is not enough information to know whether the high rates found in *Zea* are typical of all monocots, although Ref (van Overbeek, 1941) used a bioassay to estimate that *Avena* coleoptiles synthesize 13 times less auxin than *Zea*.

**Figure 3:**
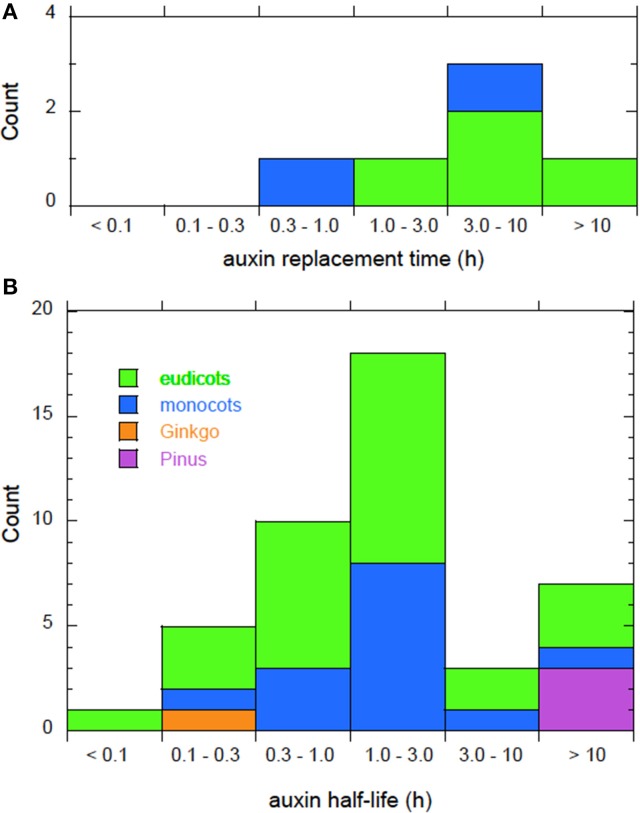
**Time scales characterizing auxin metabolism. (A)** The replacement time—the time it would take auxin biosynthesis or conjugate hydrolysis to replace the current auxin content of the plant or tissue. **(B)** The half-life—the time it would take catabolism or conjugation to reduce the current auxin content by 50%. Histograms compiled from data in Tables S1 and S2 respectively.

Auxin replacement times appear in Figure 3A and Table S1. They range from a minimum of 0.31 h to a maximum of 11 h, with a median value of 4.4 h. By this measure, too, *Zea* coleoptiles are notable, since they have the shortest replacement time of any plant tissue for which we have data. Auxin biosynthesis is one of the principle functions of the coleoptile apex, and it appears to be specialized for that role (Mori et al., 2005; Nishimura et al., 2009).

Our rates for auxin catabolism and conjugation are shown in the right half of Figure 2 and detailed in the online Supplement (Table S2). We draw on data from 24 papers covering 11 eudicots, two monocots, three species of *Pinus*, and the tree species *Ginkgo biloba*. We find that the oxIAA and IBA pathways have rates comparable to the biosynthesis rates described above, with values between 10 and 40 nM/h. Conversely, auxin conjugation shows a remarkably wide range of rates, with some values exceeding 100 μM/h. This range reflects the fact that conjugation is first order, so that the rate depends on the concentration of applied IAA. In Arabidopsis leaves, the production of IAAsp is approximately linear in applied auxin, with no sign of saturation even at a metabolic rate of 143 μM/h (Barratt et al., 1999). Thus, the highest rates of conjugation in Figure 2 are presumably artifacts that do not reflect whole-plant rates in the absence of added auxin. However, in the next section we discuss the possibility that these high rates do indeed play a functional role in plant growth and development, in cases where auxin transport produces a localized auxin accumulation. As for why the rate of oxIAA and IBA production do not exhibit similarly high values, we suggest that these pathways saturate at rates around 100 nM/h or less. Consistent with this, Sztein et al. (1995) found high rates of conjugation in seven species of seed plants with applied auxin, but did not detect any production of oxIAA.

Figure 3B and Table S2 present data on auxin half-lives. Values range over three orders of magnitude in time, with a minimum of 2.0 min, a median value of 1.7 h, and a maximum greater than 70 h. The two shortest half-lives in the set are measured in root cortex (7.5 min) and in a cell culture derived from root cortex (2.0 min), suggesting that root cortex tissue may be specialized for rapid auxin metabolism. The relevance of this observation for root system development will be discussed in the next section. The longest half-lives in the set (greater than 10 h) show that not all plant tissues can efficiently metabolize auxin, a fact that that will be discussed further in the section Auxin Metabolism and Transport.

## Auxin sinks

One of the clearest implications of Figure 2 is that the rate of auxin conjugation can exceed by orders of magnitude the other auxin metabolic rates in the plant. The high rate of auxin conjugation, in particular the formation of IAAsp, was first observed by Andreae and Good (1955), who suggested that conjugation was a mechanism for auxin detoxification. It was subsequently shown that auxin conjugation is ubiquitous among seed plants exposed to high levels of exogenous auxin (Sztein et al., 1995). However, since levels of auxin conjugates are typically low in the absence of applied auxin, the relevance of conjugation for normal plant function has been called into question (Tam et al., 2000).

**Figure 4:**
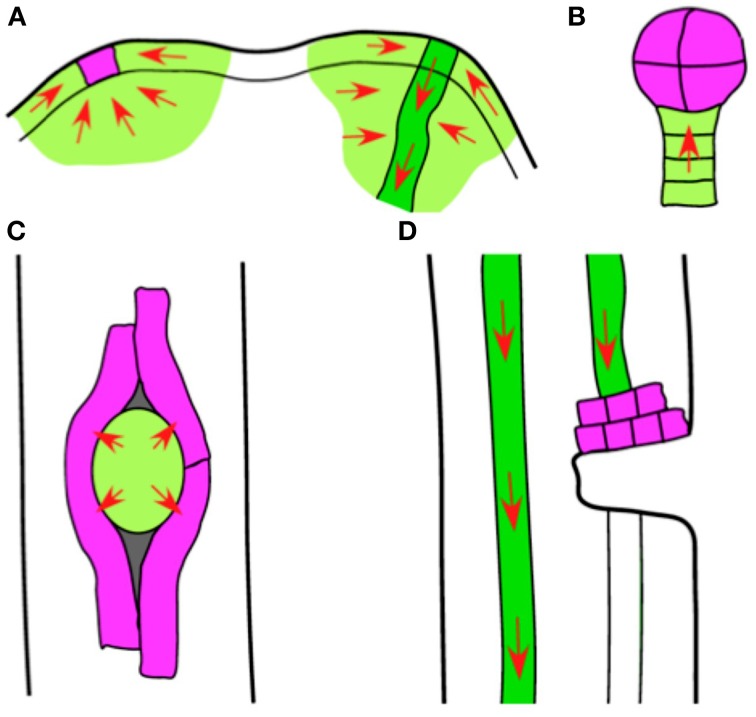
**Sketches of putative auxin sinks**. In the development of Arabidopsis and other seed plants, there are many cases where auxin transport (red arrows) drives an accumulation of auxin. **(A)** Two stages in the development of an organ primordium in the shoot apical meristem (SAM). The primordium eventually develops a provascular strand (right, dark green) that exports auxin from the SAM, supplied with auxin from many adjacent cells (light green). There is an earlier stage in primordium development (left) where all auxin transport converges on just one or a few cells (purple). **(B)** An early-stage embryo. Auxin is transported through the suspensor (light green) into the embryo (purple). **(C)** Emergence of a lateral root primordium. As the primordium (light green) grows through the root cortex and epidermis, only those outer-root cells in direct contact with the primordium (purple) show auxin-mediated gene activation. **(D)** A wounded stem. If the wound severs a vascular bundle (dark green), auxin transported through the bundle accumulates in a thin layer of cells immediately above the wound (purple).

Here we propose an endogenous role for auxin conjugation. We suggest that it is a principle pathway for catabolism (via IAAsp) and sequestration (via other conjugates) in auxin *sinks*. We define an auxin sink to be any localized group of cells that dispose of auxin principally by conjugation rather than auxin efflux. There has been little discussion of auxin sinks in the modern literature, since the prevailing idea is that auxin transport tends to drive auxin in loops or along transport pathways where catabolism is relatively unimportant (Grieneisen et al., 2007; Jones et al., 2009). However, there are several examples from plant development where the auxin transported into a region has no pathway for efficient efflux (Figure 4). We infer that much of the auxin in this case is conjugated by a small set of cells, often fewer than ~100 cells, and sometimes just one.

The best-studied case of a possible auxin sink occurs in the L1 layer of the shoot apical meristem (Figure 4A). A new organ primordium initiates as an auxin convergence point, with dozens of neighboring cells all transporting auxin toward a common center (Bayer et al., 2009; Vernoux et al., 2011). These convergence points are visible in images of fluorescently tagged PIN1 auxin efflux carriers, which form localized patches on the cell membrane and indicate the direction of auxin transport. During the earliest stages of organ initiation, cells located beneath the new primordium also transport auxin into the convergence point, although after several hours the PIN carriers change their polarity and initiate a new provascular strand.

A second example of an auxin sink is the plant embryo (Figure 4B). The Arabidopsis embryo receives auxin via PIN-mediated transport through the suspensor until the globular stage (16 or 32 cells), when an abrupt flip in PIN polarity allows auxin to exit the embryo (Friml et al., 2003; Wabnik et al., 2013). The amount of auxin that accumulates in the early-stage embryo is difficult to measure because of the small number of cells involved, but there are hints from carrot (*Daucus carota*) that the increase can be an order of magnitude or larger (Wilson et al., 1988; Ribnicky et al., 2002).

A third example of an auxin sink occurs during lateral root emergence (Figure 4C). In Arabidopsis, new lateral roots initiate in the pericycle and subsequently must penetrate the overlying cells. The developing root primordium exports an auxin signal to the cells of the outer root, but auxin-promoted gene activity is limited to those few cortical and epidermal cells that come into direct contact with the primordium (Peret et al., 2013). The limited domain of activation, typically just a few cells, suggests that auxin transported from the primordium to the outer root is rapidly metabolized to prevent it from activating genes in more distant cells. Our observation that root cortical cells metabolize auxin faster than any other tissue (see Auxin Metabolic Rates) is consistent with this model.

To this list we can add the auxin accumulation zone that forms above a wound in a stem or leaf (Figure 4D). If the wound interrupts an auxin transport stream, then PIN-mediated transport continues to pump auxin into a narrow zone of cells above the wound (Wilson et al., 1988; Kramer et al., 2008). The accumulation of auxin eventually triggers a rearrangement of the PIN polarity near the wound, but completion of this process can take several days (Sauer et al., 2006). Measurements of auxin content made above wounds show an increase of at least 2 or 3x, while measurements at the base of excised stem segments show an increase of 10–100x (Wilson et al., 1988; Nordström et al., 1991; Kramer et al., 2008).

In each of these cases, there is a developmental phase of limited duration when a small number of plant cells are effectively terminal sinks for the auxin transported through the tissue. We suggest that auxin conjugation is the endogenous pathway that prevents a runaway increase in auxin content in these cases.

The possibility that auxin conjugation plays a role in auxin sink tissues is supported by observations of the GH3 gene family, known to conjugate IAA (Staswick et al., 2005). Work on gene expression in Arabidopsis and tobacco (*Nicotiana tabacum*) routinely finds strong and rapid upregulation of GH3 family members following auxin application (Li et al., 1999; Redman et al., 2004; Goda et al., 2008). In addition, GH3 is upregulated in places where endogenous auxin is expected to accumulate, including the zone above wounds in leaves (Li et al., 1999). The general picture that emerges from this work is that auxin accumulation in a nascent sink tissue upregulates auxin conjugation, preventing auxin toxicity and limiting the size of the region subject to auxin-mediated gene activation.

The role of conjugation in an auxin sink can be further clarified with a simplified numerical example. Consider a portion of the shoot apical meristem containing 100 cells of similar size, each synthesizing auxin at a rate of 50 nM/h. We assume all this auxin is transported by PINs into a single sink cell (Figure 4A), destined to become an organ primordium. Auxin conjugation can balance transport into this sink. Since the rate of conjugation increases in proportion to the auxin concentration (Barratt et al., 1999), we can write the rate of conjugation as *c/τ*, where *c* is the auxin concentration in the sink cell and τ is a decay time, comparable to the auxin half-life, that we take to be 1.0 h (see the Supplemental Information for more discussion of this time scale). At steady-state, the balance between transport and conjugation is, (100 cells)(50 nM/h) = *c*/(1 h). This gives a steady-state concentration in the sink cell of 5.0 μM and a conjugation rate of 5.0 μM/h, both well within the range of measured values for Arabidopsis (Barratt et al., 1999; Petersson et al., 2009).

The reader may be curious whether auxin metabolic sources occur on a similarly small scale. As far as we know, they do not. For example, the shoot apical meristem does not exhibit cases where PIN transporters are coordinately arranged to point *away* from a single cell. The smallest developmentally relevant auxin source yet characterized is the lateral root primordium, which exports auxin from the pericycle to the endodermis as early as the 6-cell stage (Rybel et al., 2010). However, this set of cells is not isolated from the surrounding tissues of the stele, and much of its auxin may derive from transport rather than *de novo* biosynthesis. The smallest source of metabolically-derived auxin is the Arabidopsis embryo at the 32-cell stage, which exports auxin into the suspensor (Friml et al., 2003). The absence of smaller metabolic sources is presumably due to the low rates of auxin biosynthesis, as we discuss further in the next section.

## Auxin metabolism and transport

The survey of auxin replacement times (Table S1 and Figure 3A) allows us to directly compare the activities of auxin biosynthesis and auxin transport. Cell-to-cell auxin transport is mediated by auxin-specific carriers present on the cell membrane (Zazimalova et al., 2010). Measured transport velocities are typically ~1.5 μm/s, but may be as low as 0.3 μm/s (Kramer et al., 2011). To quantify the relative rates of transport and synthesis, we compare the time it would take a cell 15 μm long to replace all its auxin by transport to the time it would take a cell to synthesize all its auxin. The time for auxin to be renewed by transport is simply the cell length divided by the transport velocity. This will typically be ~10 s, although it may be as high as 1 min for slowly transporting tissues. The time for auxin to be replaced by biosynthesis is the replacement time (Figure 3A and Table S1), which is at least 17 min and may be hours. In other words, the time scale of auxin biosynthesis is at least an order of magnitude slower than the time scale of auxin transport.

This difference between biosynthetic and transport time scales poses a significant constraint on the spatial organization of auxin transport in the plant. A file of cells engaged in auxin transport is limited by the amount of auxin it can accumulate at its starting point. Since the amount of auxin passing through a transporting cell per unit time is an order of magnitude larger than the amount a single cell can synthesize, each file must be supplied by a source composed of dozens or hundreds of nearby cells. This is consistent with observations of auxin sources, as described in the previous section.

The mismatch in time scales may be the reason that long-range auxin transport is frequently limited to a subset of the available tissue, sometimes called an auxin *canal* (Kramer, 2004). Auxin canals are a well-studied feature of stem and leaf development, where the transporting files develop into new vascular strands (Sauer et al., 2006; Marcos and Berleth, 2014). Other examples of the spatial restriction of auxin transport include the vascular cambium of trees and the layer of cells in the root apex that carries the auxin signal during gravitropic bending (Uggla et al., 1996; Swarup et al., 2001). Indeed, spatial restriction appears to be a nearly universal feature of auxin transport in seed plants. The balance between auxin transport and biosynthesis may be a key feature in these systems.

Another strategy the plant may use to counter low biosynthesis rates is to recycle the available auxin by transporting it in a loop. Such a recycling is supposed to take place in the root meristem, where it is called a reflux loop (Blilou et al., 2005). The degree to which auxin is recycled in other tissues is unknown.

A third strategy the plant may use to counter low rates of biosynthesis is auxin conservation. Looking back to Figure 3B, recall that there is a cohort of plant tissues with a long (>10 h) auxin half-life. This demonstrates that not all plant tissues are competent to respond to high auxin concentrations with rapid increases in catabolism and conjugation. In particular, the data suggest that auxin catabolism may be inhibited in tissues specialized for long-distance auxin transport. For example, Nonhebel et al. (1985) measure the auxin half-life in the cortex and stele of maize roots as 0.12 and 21 h respectively. In the stele, where auxin undergoes transport toward the root apex, auxin catabolism is 170x slower than in the cortex. Bourbouloux and Bonnemain (1974) apply radio-labeled auxin to the apex of intact *Vicia fava* plants. After 14 h, they observe transport throughout the plant while catabolism remains relatively low. The most remarkable suppression of auxin catabolism may be the vascular cambium of trees, which is specialized to transport auxin from the shoots to the roots over a distance of many meters. The vascular cambium of Scots pine (*Pinus sylvestris*) has the slowest catabolism of any entry in Table S2, with a half-life greater than 70 h (Sundberg et al., 1994).

## Discussion

This paper presents a survey of auxin metabolic rates in plants, allowing us to analyze the balance between auxin transport and metabolism in semi-quantitative terms. In particular, we estimate that auxin metabolic sources will typically be composed of hundreds of cells, while auxin metabolic sinks may be as small as a single cell. We also find evidence that coleoptiles are among the most efficient auxin sources yet studied, while root cortex tissue is the most efficient auxin sink. These results illustrate the important insights to be gained from a quantitative approach to auxin metabolism.

The data presented here do have some limitations. In particular, they are generally measured for whole plants or excised organs, and so lack the spatial and temporal resolution that may be necessary to put gene activity in proper developmental context. Recent work on the YUCCA gene family suggests that localized auxin biosynthesis plays an important role in development (Zhao, 2008). Similarly, the existence of localized, transport-driven auxin sinks such as the early-stage embryo (Friml et al., 2003; Wabnik et al., 2013) highlights the need for improved spatial resolution of auxin catabolism and conjugation.

Complementary to the experimental approach, a second way to examine the connection between auxin metabolism and plant development will be the construction of new computer models. Most computer models of auxin action published to date do not include metabolism at all, or do so in a simplistic way with little reference to experimental observations (e.g., Bayer et al., 2009; Jones et al., 2009). The justification for this choice has been the expectation that auxin metabolic rates are low compared to auxin transport, so that the detailed localization and timing of auxin metabolism should not play a major role in development (Grieneisen et al., 2007). However, the phenotypes of metabolic mutants show clearly that auxin metabolism can have significant developmental consequences, both locally and throughout the plant (Boerjan et al., 1995; Zhao, 2008). The quantitative connection between auxin metabolism and plant development remains a rich topic for future study.

### Conflict of interest statement

The authors declare that the research was conducted in the absence of any commercial or financial relationships that could be construed as a potential conflict of interest.

## Gene names

DAO: dioxygenase for auxin oxidation
GH3: gretchen hagen 3
ILL: ILR1-like
ILR1: IAA-leucine resistant 1
PIN: pin-formed
TAA1: tryptophan aminotransferase of Arabidopsis 1
TAR: tryptophan aminotransferase related

**Chemical abbreviations**
IAA: indole-3-acetic acid
IAAsp: indole-3-acetyl aspartic acid
IBA: indole-3-butyric acid
IPyA: indole-3-pyruvic acid
NPA: naphthylphthalamic acid
oxIAA: oxindole-3-acetic acid
Trp: tryptophan.
