# Supplementary material for: Auxin metabolism rates and implications for plant development
Source: Front Plant Sci. 2015 Mar 17;6:150. doi: 10.3389/fpls.2015.00150 (PMC4362085; doi:10.3389/fpls.2015.00150)
Supplement: Supplementary file 3 [file DataSheet1.PDF]

Supplemental Information for "Auxin metabolism rates and implications for plant development", Eric M. Kramer & Ethan M. Ackelsberg.

I. Introductory notes

In this appendix we describe the procedure used to estimate the auxin metabolic rates for each entry in the supplemental tables. Section II covers biosynthesis and conjugate hydrolysis (results summarized in Table S1) and Section III covers conjugation and catabolism (Table S2).

Units. In studies on metabolism, auxin content is generally reported per gram fresh weight (g FW) of tissue. By contrast, most other research on auxin uses concentration expressed in nM or μM. In order to re-express auxin metabolic rates in units that can be compared directly with transport and signaling results, we approximate the density of plant tissue by the density of water, 1 kg per liter. This approximation should be accurate to +/- 30% (Iversen 1949; Ngonyamo-Majee *et al.* 2008). The units conversion is then 100 pg/mg FW = 571 pmol/g FW = 571 nM.

Time scales. The calculation of the auxin replacement time presented in Table S1 assumes that the auxin biosynthesis rate does not depend sensitively on the auxin concentration in the tissue. The replacement time is the auxin concentration divided by the auxin biosynthesis rate.

The calculation of auxin half-life presented in Table S2 assumes that auxin conjugation and catabolism are first order, i.e. linearly dependent on the auxin concentration (Barratt *et al.* 1999). The ratio of concentration to metabolic rate is then the exponential decay time τ, the time it would take the auxin content to be reduced to 1/e = 36.8%. This can be related to the metabolic half-life, $T_{1/2}$, the time for free auxin content to be reduced by 50%, using $T_{1/2} = \ln(2)\,\tau = 0.693\,\tau$. The half-life is the most commonly quoted time scale in papers, but the exponential decay time is easier to use in calculations since the concentration *c* of a compound being catabolized or conjugated will generally decrease like

$$c(t) = c_0 \exp(-t/\tau) \qquad \text{(S1)}$$

where c_0 is the initial concentration. For convenience, we provide both time scales in Table S2. The exponential decay time may also be estimated in cases where the balance between the auxin entering a tissue and auxin metabolism results in a steady-state auxin concentration. In that case, the rate of metabolism is $R = c/\tau$, where c is the steady-state concentration of free IAA. Thus, if the concentration of auxin in a tissue is approximately

constant, the decay time may be estimated if the conjugation or catabolism rate R is also known.

The calculation of both the replacement time and the half-life ignore the feedbacks that regulate auxin concentration in a plant tissue (Korasick *et al.* 2013; Ljung 2013), so they should be regarded as approximate.

Data extraction and curve fits. To extract numerical data from previously published figures, we used the image analysis software ImageJ (version 1.43u, http://rsb.info.nih.gov/ij). Curve fits were performed using Kaleidagraph (version 4.5, Synergy Software, Reading, PA).

II. Biosynthesis and conjugate hydrolysis

Entries are organized alphabetically by the last name of the first author.

Bialek and Cohen (1992) looks at cotyledons of germinating *Phaseolus vulgaris* (string bean) seeds. Fig. 1A shows, in the first 24 hours, approximately 50 ng of conjugated IAA is lost (from 215 to 165 ng IAA equiv./cotyledon), presumably converted to IAA. The average fresh weight of a cotyledon during this period is 510 mg (Fig. 3a). This gives a synthesis rate of (50 ng/d)/(510 mg FW) = 560 nM/d = 23.3 nM/h.

Fig. 1B shows that the average concentration of free IAA in this period is 23 ng/gFW = 131 nM.

Epstein et al. (1980), Figure 3, has a complete diagram of the pool sizes and fluxes of auxin metabolites in a germinating *Zea mays* kernel. The pool of free IAA is 308 pmol. Synthesis of IAA from Trp is 15 pmol/h and from conjugates is 80 pmol/h. Using the mass of the kernel, 0.23 g, (pg. 417) to convert to concentration, this becomes an auxin concentration of 1.34 μM and synthesis rates of 65 nM/h from Trp and 348 nM/h from conjugates.

The auxin replacement time is 1.34 μM/(0.065+0.348 μM/h) = 3.2 h

Liu et al. (2011), Fig. 7A, shows the endogenous distribution of IAA in *Solanum lycopersicum* (tomato) hypocotyls. The hypocotyls are sectioned into a “Top” portion that includes the cotyledons and hook region, followed by consecutive 6 mm hypocotyl segments. Xing Liu generously provided us with the raw data used to make Fig. 7A. This shows that the auxin content in each 6 mm hypocotyl segment was approximately 0.1 ng, and the mass of the “top” segment was about 26 mg fresh weight.

The flux of auxin exiting the "Top" segment can be estimated if we know the speed of auxin transport through the hypocotyl. Their Fig. 6 allows us to estimate the speed of auxin transport as (14 mm)/(3 h) = 4.67 mm/h. Then, the rate of auxin biosynthesis in the "Top" segment is equal to the flux of auxin downward through the hypocotyl, (.1 ng/6 mm)(4.67 mm/h) = 0.78 ng/h. Dividing by the fresh weight of the synthesizing tissues (the "Top" section, mass 26 mg), gives 3 pg/h/gFW = 17 nM/h.

The concentration of free auxin in the "Top" segment is 17 ng/gFW = 97 nM.

Ljung et al. (2001), Figure 5, shows the auxin content of *Arabidopsis thaliana* leaves. Control leaves have an approximately constant auxin content of 10 pg/mg FW. Between 8 and 16 hours, the NPA-treated leaves develop an extra 12 pg/mg FW (increasing from 10 to 22 pg/mg FW). This is a biosynthesis rate of (12 pg/mg FW)/8 h = 8.56 nM/h.

The average concentration of auxin in treated leaves at this time is 16 pg/mgFW = 91 nM.

Mori et al. (2005) studies auxin content and export in *Zea mays* coleoptiles. Fig. 4, shows that 424 pg/h are effluxed from the excised 2 mm apex of a maize coleoptile in the first 3.5 h. From work by the same research group (Nishimura *et al.* 2006) (pg. 1430), we know that the mass of a 2 mm apex is approximately 1.9 mg FW. Combining these gives us a synthesis rate of 223 pg/h/mgFW = 1.27 μM/h.

Mori et al., Fig. 2, tells us how much free IAA is in the apex. The first mm contains 82 pg IAA and the second mm contains 50 pg IAA. This gives a concentration of 69 pg/mgFW = 392 nM. Nishimura2009 (Nishimura *et al.* 2009) shows that this synthesis is principally from tryptophan, not conjugates (see especially their supplemental figure 1).

Strader et al. (2010) studies Arabidopsis seedlings. Fig. 1C shows, after 1 h incubation in 10 μM label-IBA, the seedlings have 5.8 ng/gFW = 33 nM label-IAA. Thus we have a whole-seedling auxin biosynthesis rate, from IBA only, of 33 nM/h.

Tao et al. (2008), Fig. 3B, shows auxin content for whole Arabidopsis seedlings. Seedlings transferred from continuous light to shade for 1 h increase in auxin content from 25 to 38 ng/gFW (we have estimated the first number using the control experiment). This corresponds to a synthesis rate of 13 ng/g FW/h = 74 nM/h. As the increase in auxin content is inhibited in *taa1* mutants, biosynthesis proceeds from tryptophan.

The average seedling concentration is 31.5 pg/mg FW = 180 nM.

II. Conjugation and catabolism

Entries are organized alphabetically by the last name of the first author.

Andreae and van Ysselstein (1960), pg. 227, describes an experiment where excised *Pisum sativum* (pea) root tips are incubated for 2 h in 100 μM IAA, then transferred to a moist chamber. Within 4 h, 70% of the free IAA in the tissue is converted to IAAsp. This corresponds to a decay time of 3.3 h. They use an early method for the detection of auxin metabolites, but their results are consistent with more modern techniques.

Atzmon et al. (1996) apply label-IAA to the shoot apex of a *Pinus pinea* (stone pine) seedling, with a 15 cm tap root. Fig. 3 shows that label-IAA enters the root system before 6 hrs. They say on pg. 15 that, after 48 h, 33% of the IAA in the root system is still present as free IAA. This is a decay time of 43 h.

Barratt et al. (1999), Fig. 1B, shows Arabidopsis leaves excised from plants 3-4 weeks after germination and subject to various concentrations of applied IAA. The figure shows the production of several conjugates after 24 hours, with the majority being IAAsp. At the largest value, 500 μM applied IAA, the leaves make 600 ng IAA equiv. per mg FW tissue. This is a conjugation rate of (600 ng/mg FW)/(24 h) = 143 μM/h. Other values, at smaller applied concentrations of IAA, may be calculated in the same way.

Bourbouloux and Bonnemain (1974) considers intact *Vicia faba* plants, plants, ~27 cm in length from root to shoot apex (according to the sketch accompanying their Figs. 2-5). Label-IAA applied to shoot apex at time 0.

Fig. 2-5 show chromatograms of label from stem, primary root, and lateral roots. 14 h after application, most of the label is still in the form of free IAA.

Delbarre et al. (1994) studies suspensions of leaf protoplasts isolated from *Nicotiana tabacum* (tobacco). Fig 7 shows an uptake experiment where 5.0 μM label-IAA is applied at time 0. The free IAA in the protoplasts rises to a plateau of ~ 7 μM at 40 min. Between 40 and 180 minutes, the concentration of metabolites continue to

increase linearly from 3 μM to 11 μM. This is a metabolic rate of 3.4 μM/h. Chromatography shows large amounts of IAA conjugates among the metabolites.

Edwards and Goldsmith (1980) studies auxin uptake in 1 mm slices of *Zea mays* coleoptiles. On page 463, they say that auxin taken up by maize coleoptile sections is still mostly (67 to 77%) in the form of free IAA after 1 h. The exponential decay rate in this tissue is then 2.5 h to 3.8 h. In Table S2 we use the average value, 3.2 h.

Epstein et al. (1980) conducts a thorough study of auxin metabolism in germinating kernels of *Zea mays*. They add a low concentration of label-IAA to a split kernel, and measure turnover. In the endosperm of the kernel, they measure a half-life of 3.2 h (their Table I), equivalent to an exponential decay time of 4.6 h.

Fig.3 shows the pool of free IAA is 308 pmol. Using the mass of the kernel, 0.23 g, (pg. 417) to convert to concentration, this becomes an auxin concentration of 1.34 μM

To estimate the metabolic flux, divide the concentration by the exponential decay time.

Heloir et al. (1996) studies root induction in excised shoots of *Juglans regia* (walnut). See Fig. 2A and B. In the first 12 hours after culture on root-inducing medium, free IAA concentration averaged 3.6 μM in the basal third of the shoot. The concentration of IAAsp increased from 7.5 μM to 22 μM in the same 12 hours, giving a conjugation rate of 1.2 μM/h.

Loper and Spanswick (1991) studies suspension-cultured *Glycine max* (soybean) callus cells derived from cortical root tissue. They apply label-IAA and chromatograph the cell contents (Fig. 2). On page 187 they say, "After 1 min of uptake (Fig. 2B), only 70% of the radioactivity is present as IAA and at least two other metabolites (aqueous phase) have formed." This corresponds to an approximate exponential decay time of 2.8 min = 0.047 h (this value is only approximate, as the free auxin content of the cells is increasing due to auxin influx during the first minute).

We can also estimate the metabolic rate in these cells. According to Fig. 3, at the 60 s mark, the concentration of label is about 5.3 pmol/mg DW (DW = dry weight). On page 190, they note that 1 mg DW corresponds to approximately 40 mg FW of cells. The concentration of label is thus 5.3 pmol/(40 mg FW) = 132 nM. Since 30% of this label is

metabolized after the first minute, the rate is (0.3)(132 nM)/(1 min) = 2.38 μM/h. The concentration of free IAA in cells at the 1 min mark is (0.7)(132 nM) = 92 nM.

Ludwig-Muller and Epstein (1991) studied conversion of ^{14}C-IAA to ^{14}C-IBA in 2 mm excised sections of *Zea mays* seedlings, 8 dag. Their Table I shows label per gram fresh weight in the form of IBA after 1 h incubation in 0.54 μM label-IAA. The label-IAA has activity 1.85 TBq/mol (pg. 765), permitting conversion from Bq/(g FW)/h to μM/h. For example, according to Table I, root segments of variety Hazera 224 convert 28 Bq/g FW/h to IBA. This is 0.015 μM/h.

Maldiney and Miginiac (1982) studies intact *Solanum lycopersicum* (tomato) seedlings. The focus on the rapid transport of label-IAA through the phloem after application of IAA to the shoot apex. Fig. 3 shows the distribution of label throughout the plant at the 10 minute mark, and Fig. 4 at the 6 hour mark.

Let's first focus on the hypocotyl segment just below the cotyledons. At 10 minutes, this has a total of 350 cpm, 80% of which chromatographs as free IAA, for a total of 280 cpm IAA. At 6 hours, this segment has a total 3110 cpm, with 6% free IAA and 80% IAAsp, or 187 cpm IAA and 2488 cpm IAAsp respectively. We thus have a free IAA content with mean value 234 cpm, and an IAAsp production rate of 2488 cpm/(6 h) = 415 cpm/h. As described in Section I of this appendix, at steady-state the conjugation rate = (free IAA concentration)/(exponential decay time), so the decay time is 0.56 h.

The same approach gives similar results for most other stem segments, but the shoot apex is an exception, with a decay time of 14.7 h. It's possible that metabolism in the shoot apex is saturated, since this is where the high concentration of label-IAA is applied.

Malloch and Osborne (1976) studies 5 dag coleoptile and mesocotyl segments from *Avena sativa* (oat) and *Zea mays* (maize).

Avena: Fig. 7 shows the distribution of free IAA and metabolites following the application of 5 μM label-IAA for the first 2 h. We extracted the data from the figures and then fit a decaying exponential to the label left as free IAA. Curve fits give exponential decay times of 2.0 and 2.5 h for the coleoptile and mesocotyl data respectively.

Zea: Fig. 6 shows the distribution of free IAA and metabolites following the application of 5 μM label-IAA. The amount of IAA does not vary much over the duration of the experiment, so a half-life cannot be determined.

Mori et al. (2005) studies auxin content and export in *Zea mays* coleoptiles. Table 1 shows that the "total" IAA (i.e. conjugated plus free) in the apical 2 mm of the coleoptile increases from 2243 pg to 2761 pg between 0.5 h and 3.5 h after excision. Since Table 1 also shows that the free IAA is approximately constant over this time, we conclude that the conjugated IAA content increases by 518 pg in 3.0 h. The same research group (Nishimura et al. 2006) (pg. 1430) reports that the mass of a 2 mm apex is approximately 1.9 mg FW. We thus estimate a conjugation rate of 0.52 μM/h.

The free IAA content over this time interval averages 116 pg per tip (Table 1). This gives a concentration of (116 pg)/(1.9 mg FW) = 349 nM.

Morris et al. (1969) applies label-auxin to apical buds of intact *Pisum sativum* (pea) plants, 12-14 dag, then uses chromatography to identify metabolites.

Fig. 6, shoot apex. Between 3 and 4.5 h, the IAAsp increases by 0.81 x 10^5 dpm. Average IAA label over same period is 1.5 x 10^5 dpm. Thus, the exponential decay time τ = 1.5/(0.81/1.5h) = 2.8 h.

Fig. 7a, internode 3. Between 3 and 4.5 h, IAAsp increases by 1.1 x 10^3 dpm. The average IAA is 1.55 x 10^3 dpm. Thus, τ = 1.55/(1.1/1.5h) = 2.1 h.

Fig. 7b, internode 2. Between 3 and 4.5 h, IAAsp increases by 0.69 x 10^3 dpm. The avg. IAA concentration is 1.3 x 10^3 dpm. Thus, τ = 1.3/(0.69/1.5h) = 2.8 h.

Fig. 8a. internode 1. Between 3 and 6h, IAAsp increases by 0.94 x 10^3 dpm and a second metabolite increases by 0.27 x 10^3 dpm. The average IAA is 0.29 x 10^3 dpm. The τ = .29/(1.21/3 h) = 0.72 h.

Fig. 8b. roots. Between 3h and 9h, IAAsp increases by 3.9 x 10^3 dpm and a second metabolite increases by 2.1 x 10^3 dpm. Over the same time, IAA averages about 0.65 x 10^3 dpm. Thus, τ = .65/(6.0/6 h) = 0.65 h.

Nonhebel et al. (1983) studies metabolism of label-IAA in 20 mm segments of *Zea mays* roots. Their Table 3 shows the results of four experiments, with concentrations of exogenous label-IAA 0.1 μM, 1 μM, 10 μM, and 100 μM. The values in this table can be converted to metabolic rates as follows. The table lists label uptake after 2 h in 0.1 μM as 2.3 pmol/segment. The average mass of a segment is 17.6 mg FW (pg. 133), so this is a label concentration of 131 nM. After 2 h, 25% of the label is still present as IAA, giving a concentration for free IAA of 33 nM. The remaining 75% has been metabolized, for a rate of 49 nM/h. Assuming a steady-state has been reached, the exponential decay time is

approximately (33 nM)/(49 nM/h) = 0.67 h. The other experiments in the table are analyzed in the same way.

Nonhebel et al. (1985c) studies metabolism of label-IAA in segments of *Zea mays* coleoptiles. Their Table 3 shows very similar decay rates for three different concentrations of exogenous IAA – 1 μM, 10 μM, and 100 μM. After a 2 h incubation, the fraction of label in the sections remaining as free IAA is (57 ± 1)%, (45 ± 3)%, and (58 ± 5)% respectively. This corresponds to a decay time of 3.6 h, 2.5 h, and 3.7 h respectively.

Nonhebel et al. (1985r) study metabolism of label-IAA in segments of *Zea mays* roots. In this case, they take advantage of the fact that the cortex of the root can be physically separated from the stele, allowing them to measure metabolism is each portion separately.

Their Table 1 shows the results of root segments incubated in 1 μM label-IAA. After 2 h, they found the stele had metabolized only 1% to 6% of the label while the cortex had metabolized 91% to 92%. These can be converted into decay times in the following way. In their first replicate on cortex tissue, they observe uptake of 39 Bq per segment. As 8% of this label is present as IAA, we have 3.1 Bq IAA per segment. The remainder has been metabolized, at a rate of 18 Bq/h. Assuming a steady-state, this is a metabolic decay time of (3.1 Bq)/(18 Bq/h) = 0.17 h. Repeating this calculation for each replicate, we find an average decay time in the cortex of 0.18 h and lower bound on the decay time in the stele of 31 h.

Nordstrom and Eliasson (1991a) studies the endogenous levels of auxin and metabolites in stem cuttings of *Pisum sativum* (pea). In Fig. 4b, we see that the IAAsp content in the stem base increases from 17 ng/g FW on day 0 to 120 ng/g FW on day 3. This is a rate for conversion of IAA to IAAsp of 8.3 nM/h.

Nordstrom et al. (1991b) study the effects of exogenous IAA and IBA on stem cuttings of *Pisum sativum* (pea). The IAAsp synthesis rate can be calculated as for Ref. (Nordström & Eliasson 1991a), by the same group.

Fig. 3 shows the accumulation of IAAsp in the stem base. For stems treated with IAA, the largest synthesis rate occurs between days 2 and 3 and is 435 nM/h. For stems treated with IBA, the largest synthesis rate occurs between days 1 and 2 and is 39 nM/h.

Oetiker and Aeschbacher (1997) study auxin uptake and metabolism is suspension culture cells of *Hyoscyamus muticus* (henbane). Fig. 8A shows the levels of IAA and IAAsp after addition of 30.6 μM IAA to the medium at time 0. At 0.6 h, the cells contain approximately 1.8 μM IAAsp, giving a metabolic rate of 3 μM/h. Over this time interval, the cells average about 0.6 μM IAA.

Ostin et al. (1998) presents a thorough analysis of auxin metabolism in Arabidopsis plants.

Fig. 1, top panel, shows the distribution of auxin metabolites in soil-grown plants picked and supplied with 5 μM label-IAA. After 4 h, there are approximately equal amounts of IAA and its summed metabolites. Assuming an approximate auxin steady-state during the first 4 h, we can estimate a decay time of 4 h (see the discussion of time scales in Sec. I).

Fig. 3 shows plants grown in liquid culture with 5 μM IAA added at the start of the experiment. After 2 h, there are approximately twice as many auxin metabolites as there is auxin. Since the auxin content appears to be approximately constant between 2 h and 4h, we approximate this as an auxin steady-state and estimate a decay time of 1 h.

Peer et al. (2013) applies label-IAA (20 Ci/mmol) to excised Arabidopsis root apices and counts label-oxIAA. Fig. 2R shows that label-oxIAA, measured in sets of ten 2 mm root segments, increases from 200 to 900 dpm. If we approximate the volume of the root segment as a cylinder with radius 60 microns and length 2mm, then this is a rate of oxIAA production of 18 nM/h.

Sundberg et al. (1994), Figure 5C, shows that 80% of label-IAA, fed to *Pinus sylvestris* (Scots pine) shoots, is still in the form of IAA after 4 days. This would be a half life of more than one week.

Sztein et al. (1995) measures metabolism in a wide range of plant species. In this paper we only consider their data for seed plants, shown in their Fig. 4.

Excised tissues from seven seed plant species are immersed in 154 μM radio-label IAA for 22 hours, and chromatography techniques used to quantify many of the most abundant metabolites.

For example, in Arabidopsis leaves, most of the label is metabolized after 22 h, with the majority identified with IAAsp and other conjugates (Fig. 4). The total metabolized label is 11 μg IAA equivalent/g FW, corresponding to a rate of 2.85 μM/h.

References

Andreae, W. A. and M. W. H. Van Ysselstein (1960). Studies on 3-Indoleacetic Acid Metabolism. VI. 3-Indoleacetic Acid Uptake and Metabolism by Pea Roots and Epicotyls. *Plant Physiology* 35: 225-232.

Atzmon, N., Z. Wiesman and J. Van Staden (1996). The effect of zeatin and iso-pentenyladenine on IAA transport from the shoot of the root of Pinus pinea seedlings. *Plant Growth Regulation* 19: 13-18.

Barratt, N. M., W. Dong, D. A. Gage, V. Magnus and C. D. Town (1999). Metabolism of exogenous auxin by Arabidopsis thaliana: Identification of the conjugate Nα-(indol-3-ylacetyl)-glutamine and initiation of a mutant screen. *Plant Physiology* 105: 207-217.

Bialek, K. and J. D. Cohen (1992). Amide-Linked Indoleacetic Acid Conjugates May Control Levels of Indoleacetic Acid in Germinating Seedlings of Phaseolus vulgaris. *Plant Physiology* 100: 2002-2007.

Bourbouloux, A. and J.-L. Bonnemain (1974). Transport, distribution et metabolisme de l'auxine dans la racine de Vicia faba L. apres application de [14-C]AIA ou de [3-H]AIA sur le bourgeon. *Planta* 119: 169-182.

Delbarre, A., P. Muller, V. Imhoff, J.-L. Morgat and H. Barbier-Brygoo (1994). Uptake, accumulation and metabolism of auxins in tobacco leaf protoplasts. *Planta* 195: 159-167.

Edwards, K. L. and M. H. M. Goldsmith (1980). pH-Dependent Accumulation of Indoleacetic Acid by Corn Coleoptile Sections. *Planta* 147: 457-466.

Epstein, E., J. D. Cohen and R. S. Bandurski (1980). Concentration and Metabolic Turnover of Indoles in Germinating Kernels of Zea mays L. *Plant Physiology* 65: 415-421.

Heloir, M.-c., C. Kevers, J.-F. Hausman and T. Gaspar (1996). Changes in the concentrations of auxins and polyamines during rooting of in-vitro-propagated walnut shoots. *Tree Physiology* 16: 515-519.

Iversen, J. (1949). Determinations of the specific gravity of the roots of swamp, meadow and dry-soil plants. *Oikos* 1: 1-5.

Korasick, D. A., T. A. Enders and L. C. Strader (2013). Auxin biosynthesis and storage forms. *Journal of Experimental Botany* 64: 2541-2555.

Liu, X., J. D. Cohen and G. Gardner (2011). Low-Fluence Red Light Increases the Transport and Biosynthesis of Auxin. *Plant Physiology* 157: 891-904.

Ljung, K. (2013). Auxin metabolism and homeostasis during plant development. *Development* 140: 943-950.

Ljung, K., R. Bhalerao and G. Sandberg (2001). Sites and homeostatic control of auxin biosynthesis in *Arabidopsis* during vegetative growth. *Plant Journal* 28: 465-474.

Loper, M. and R. Spanswick (1991). Auxin transport in suspension-cultured soybean root cells: I. Characterization. *Plant Physiology* 96: 184-191.

Ludwig-Muller, J. and E. Epstein (1991). Occurrence and in Vivo Biosynthesis of Indole-3-Butyric Acid in Corn (Zea mays L.). *Plant Physiology* 97: 765-770.

Maldiney, L. S. and E. Miginiac (1982). Some characteristics of a fast movement of auxin in intact tomato seedlings (Lycopersicon esculentum). *Physiologia Plantarum* 55: 361-370.

Malloch, K. R. and D. J. Osborne (1976). Auxin and Ethylene Control of Growth in Seedlings of Zea mays L. and Avena sativa L. *Journal of Experimental Botany* 27: 992-1003.

Mori, Y., T. Nishimura and T. Koshiba (2005). Vigorous synthesis of indole-3-acetic acid in the apical very tip leads to a constant basipetal flow of the hormone in maize coleoptiles. *Plant Science* 168: 467-473.

Morris, D. A., R. E. Briant and P. G. Thomson (1969). The transport and metabolism of 14C-labelled indoleacetic acid in intact pea seedlings. *Planta* 89: 178-197.

Ngonyamo-Majee, D., R. D. Shaver, J. G. Coors, D. Sapienza, C. E. S. Correa, J. G. Lauer and P. Berzaghi (2008). Relationships between kernel vitreousness and dry matter degradability for diverse corn germplasm I. Development of near-infrared reflectance spectroscopy calibrations. *Animal Feed Science and Technology* 142: 247-258.

Nishimura, T., Y. Mori, T. Furukawa, A. Kadota and T. Koshiba (2006). Red light causes a reduction in IAA levels at the apical tip by inhibiting de novo biosynthesis from tryptophan in maize coleoptiles. *Planta* 224: 1427-1435.

Nishimura, T., H. Nakano, K.-i. Hayashi, C. Niwa and T. Koshiba (2009). Differential Downward Stream of Auxin Synthesized at the Tip Has a Key Role in Gravitropic Curvature via TIR1/AFBs-Mediated Auxin Signaling Pathways. *Plant and Cell Physiology* 50: 1874-1885.

Nonhebel, H. M., A. Crozier and J. R. Hillman (1983). Analysis of [C-14] indole-3-acetic acid metabolites from the primary roots of Zea mays seedlings using reverse-phase high-performance liquid chromatography. *Physiologia Plantarum* 57: 129-134.

Nonhebel, H. M., J. R. Hillman, A. Crozier and M. B. Wilkins (1985c). Metabolism of [C-14] Indole-3-Acetic Acid by Coleoptiles of Zea mays L. *Journal of Experimental Botany* 36: 99-100.

Nonhebel, H. M., J. R. Hillman, A. Crozier and M. B. Wilkins (1985r). Metabolism of [C-14] indole-3-acetic acid by the cortical and stelar tissues of Zea mays L. roots. *Planta* 164: 105-108.

Nordström, A.-C. and L. Eliasson (1991a). Levels of endogenous indole-3-acetic acid and indole-3-acetylaspartic acid during adventitious root formation in pea cuttings. *Physiologia Plantarum* 82: 599-605.

Nordström, A.-C., F. A. Jacobs and L. Eliasson (1991b). Effect of Exogenous Indole-3-Acetic Acid and Indole-3-Butyric Acid on Internal Levels of the Respective Auxins and Their Conjugation with Aspartic Acid during Adventitious Root Formation in Pea Cuttings. *Plant Physiology* 96: 856-861.

Oetiker, J. H. and G. Aeschbacher (1997). Temperature-Sensitive Plant Cells with Shunted Indole-3-Acetic Acid Conjugation. *Plant Physiology* 114: 1385-1395.

Ostin, A., M. Kowalyczk, R. P. Bhalerao and G. Sandberg (1998). Metabolism of Indole-3-Acetic Acid in Arabidopsis. *Plant Physiology* 118: 285-296.

Peer, W. A., Y. Cheng and A. Murphy (2013). Evidence of oxidative attenuation of auxin signalling. *Journal of Experimental Botany* 64: 2629-2639.

Strader, L. C., A. H. Culler, J. D. Cohen and B. Bartel (2010). Conversion of Endogenous Indole-3-Butyric Acid to Indole-3-Acetic Acid Drives Cell Expansion in Arabidopsis Seedlings. *Plant Physiology* 153: 1577-1586.

Sundberg, B., H. Tuominen and C. H. A. Little (1994). Effects of the indole-3-acetic acid (IAA) transport inhibitors N-1-naphthylphthalamic acid and morphactin on endogenous IAA dynamics in relation to compression wood formation in 1-year-old *Pinus sylvestris* shoots. *Plant Physiology* 106: 469-476.

Sztein, A. E., J. D. Cohen, J. P. Slovin and T. J. Cooke (1995). Auxin Metabolism in Representative Land Plants. *American Journal of Botany* 82: 1514-1521.

Tao, Y., J.-L. Ferrer, K. Ljung, F. Pojer, F. Hong, J. A. Long, L. Li, J. E. Moreno, M. E. Bowman, L. J. Ivans, et al. (2008). Rapid Synthesis of Auxin via a New Tryptophan-Dependent Pathway Is Required for Shade Avoidance in Plants. *Cell* 133: 31-32.
